# Supplementary material for: Plasma Inflammatory Proteome Profile in a Cohort of Patients with Recurrent Vulvovaginal Candidiasis in Kenya
Source: J Fungi (Basel). 2024 Sep 6;10(9):638. doi: 10.3390/jof10090638 (PMC11433550; doi:10.3390/jof10090638)
Supplement: Supplementary file 1 [file jof-10-00638-s001.zip › Supplementary Figures.pptx]

## Slide 1
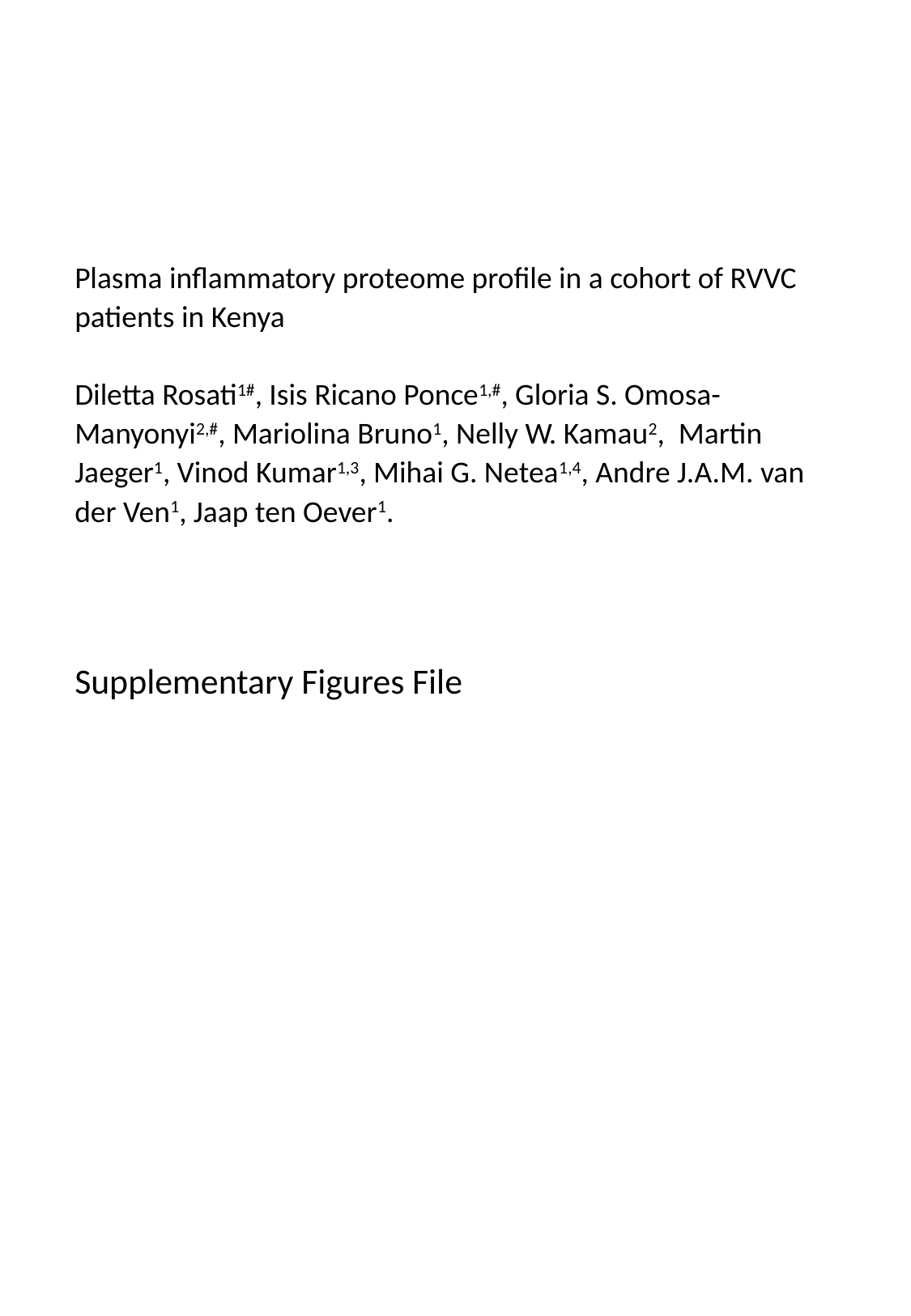

# Plasma inflammatory proteome profile in a cohort of RVVC patients in Kenya   Diletta Rosati1#, Isis Ricano Ponce1,#, Gloria S. Omosa-Manyonyi2,#, Mariolina Bruno1, Nelly W. Kamau2, Martin Jaeger1, Vinod Kumar1,3, Mihai G. Netea1,4, Andre J.A.M. van der Ven1, Jaap ten Oever1.
Supplementary Figures File

## Slide 2
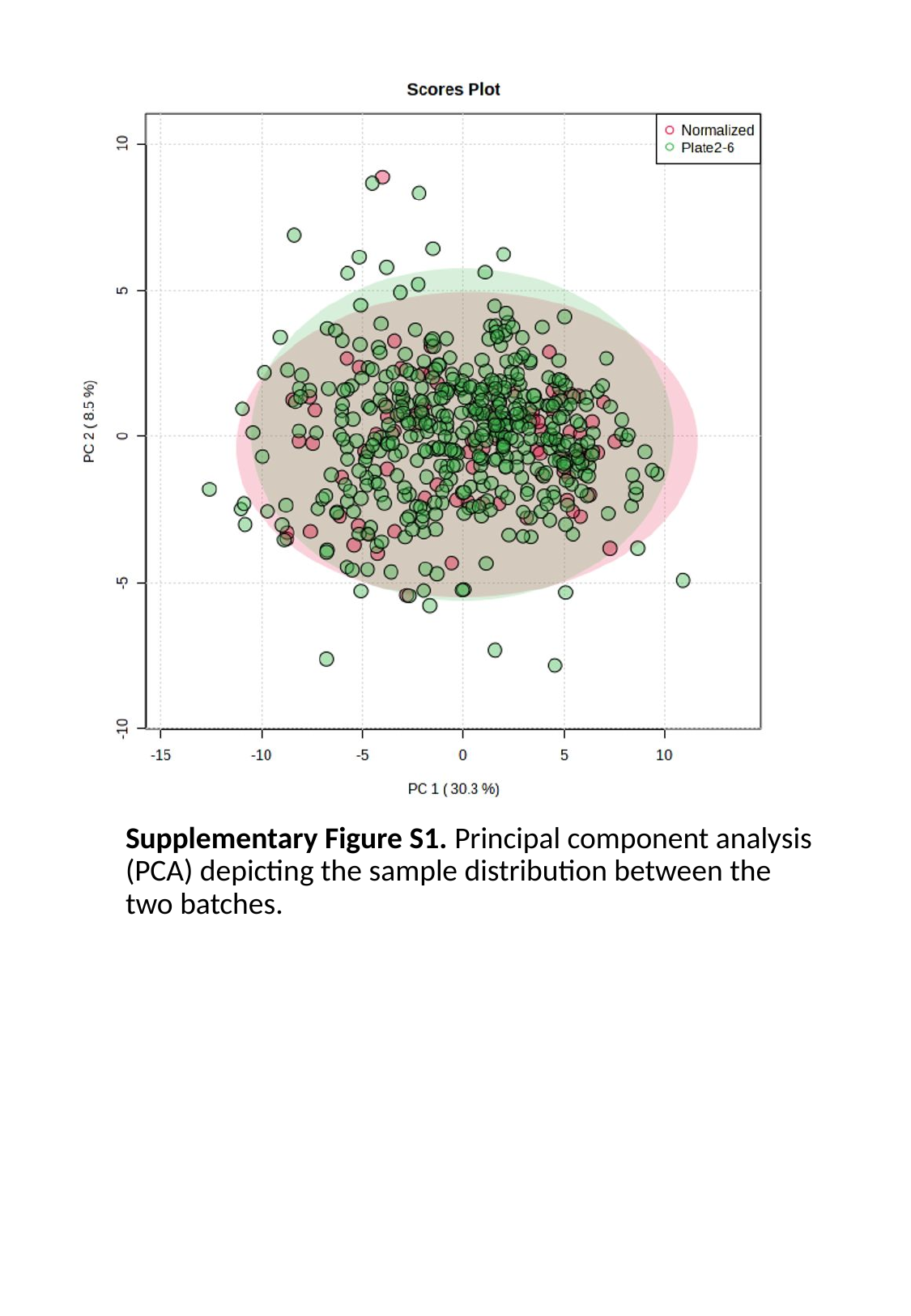

# Supplementary Figure S1. Principal component analysis (PCA) depicting the sample distribution between the two batches.

## Slide 3
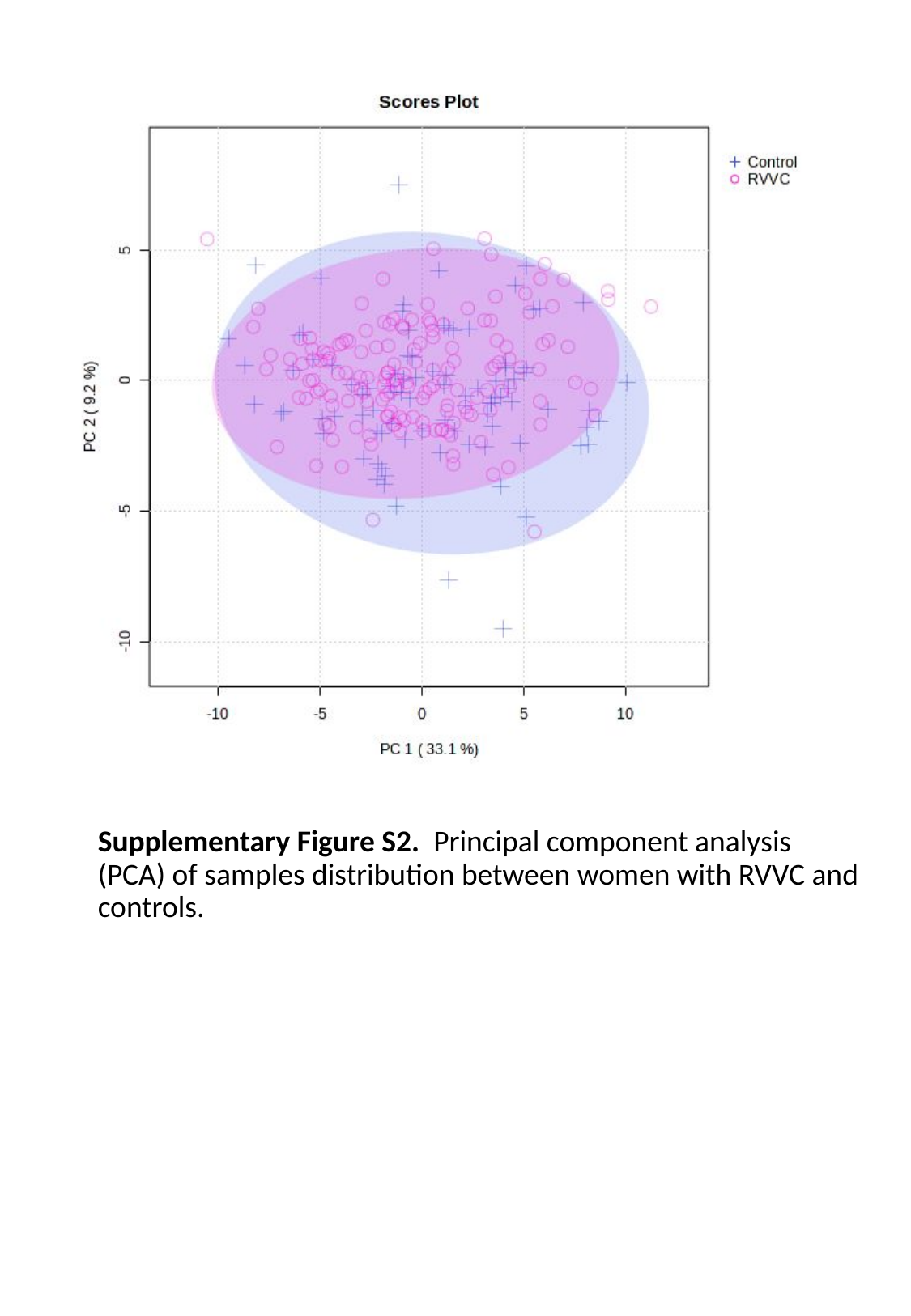

# Supplementary Figure S2. Principal component analysis (PCA) of samples distribution between women with RVVC and controls.

## Slide 4
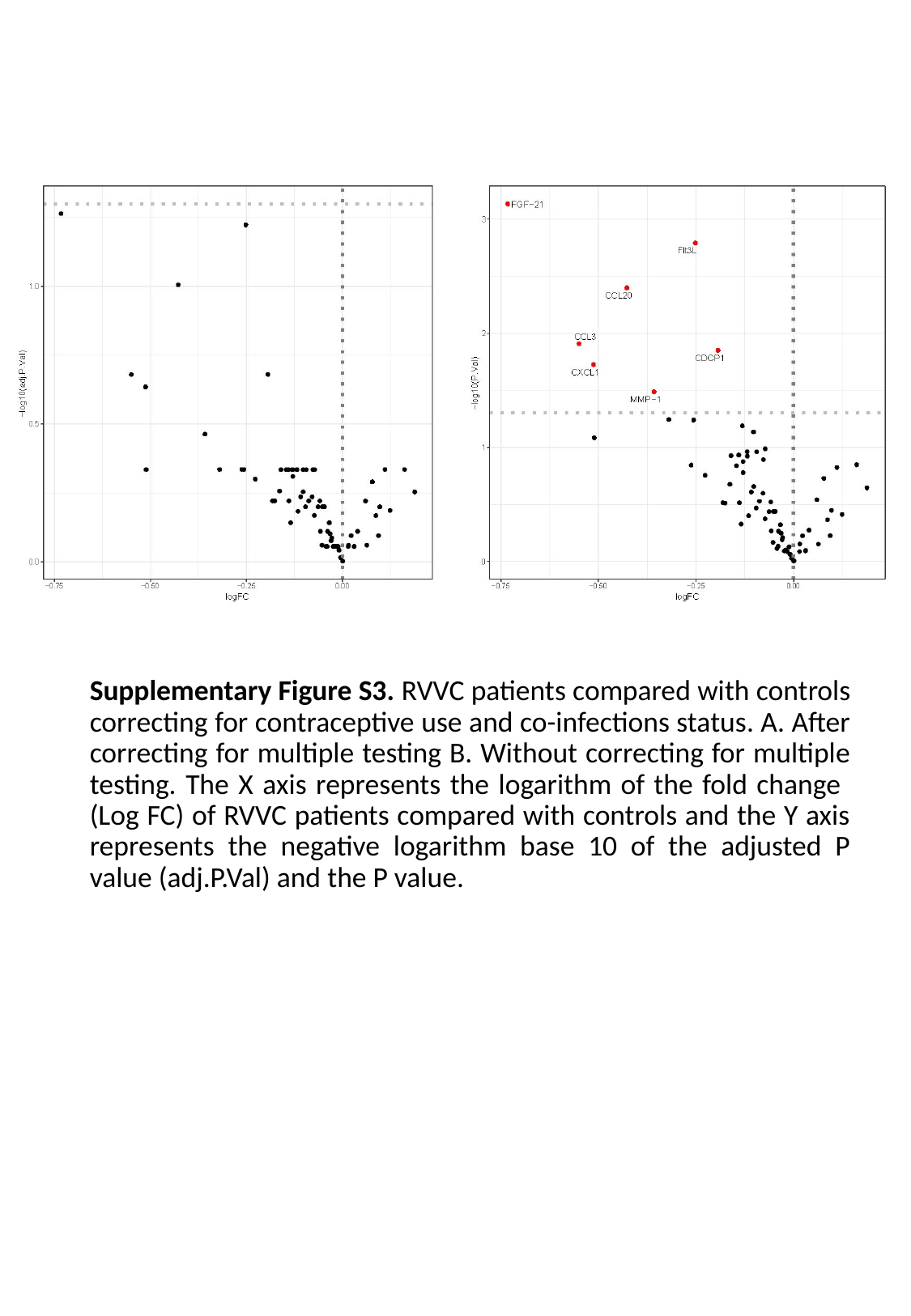

# Supplementary Figure S3. RVVC patients compared with controls correcting for contraceptive use and co-infections status. A. After correcting for multiple testing B. Without correcting for multiple testing. The X axis represents the logarithm of the fold change (Log FC) of RVVC patients compared with controls and the Y axis represents the negative logarithm base 10 of the adjusted P value (adj.P.Val) and the P value.
